# Supplementary material for: Modeling glioblastoma heterogeneity as a dynamic network of cell states
Source: Mol Syst Biol. 2021 Sep 16;17(9):e10105. doi: 10.15252/msb.202010105 (PMC8444284; doi:10.15252/msb.202010105)
Supplement: Supplementary file 6 — Source Data for Figure 5 [file MSB-17-e10105-s004.zip › Figure5A_sourcedata/GSEA_3071/nosigdb_stateI.GseaPreranked.1622795145664/VANGRONINGEN_ADRN.html]

Details for gene set VANGRONINGEN\_ADRN[GSEA]

|  || Dataset | state13071 |
| Phenotype | NoPhenotypeAvailable |
| Upregulated in class | na\_neg |
| GeneSet | VANGRONINGEN\_ADRN |
| Enrichment Score (ES) | -0.56259435 |
| Normalized Enrichment Score (NES) | -3.1878526 |
| Nominal p-value | 0.0 |
| FDR q-value | 0.0 |
| FWER p-Value | 0.0 |
Table: GSEA Results Summary

  

Fig 1: Enrichment plot: VANGRONINGEN\_ADRN      
 Profile of the Running ES Score & Positions of GeneSet Members on the Rank Ordered List

  

| PROBE | GENE SYMBOL | GENE\_TITLE | RANK IN GENE LIST | RANK METRIC SCORE | RUNNING ES | CORE ENRICHMENT || 1 | MIAT |  |  | 41 | 1.422 | -0.0008 | No |
| 2 | SYT1 |  |  | 155 | 0.906 | -0.0273 | No |
| 3 | SEPT6 |  |  | 193 | 0.841 | -0.0316 | No |
| 4 | NGRN |  |  | 226 | 0.776 | -0.0349 | No |
| 5 | TIAM1 |  |  | 341 | 0.636 | -0.0639 | No |
| 6 | L1CAM |  |  | 369 | 0.617 | -0.0670 | No |
| 7 | GLRX |  |  | 485 | 0.524 | -0.0972 | No |
| 8 | HEY1 |  |  | 497 | 0.517 | -0.0964 | No |
| 9 | RNF144A |  |  | 730 | 0.402 | -0.1626 | No |
| 10 | NCOA7 |  |  | 755 | 0.396 | -0.1666 | No |
| 11 | C4orf48 |  |  | 772 | 0.392 | -0.1682 | No |
| 12 | RPS6KA2 |  |  | 817 | 0.376 | -0.1784 | No |
| 13 | SLIT3 |  |  | 911 | 0.348 | -0.2034 | No |
| 14 | MAP1B |  |  | 1198 | 0.289 | -0.2868 | No |
| 15 | POPDC3 |  |  | 1259 | 0.275 | -0.3025 | No |
| 16 | ATL1 |  |  | 1292 | 0.269 | -0.3099 | No |
| 17 | GATA3 |  |  | 1417 | -0.250 | -0.3450 | No |
| 18 | ANK2 |  |  | 1431 | -0.251 | -0.3469 | No |
| 19 | MMD |  |  | 1511 | -0.258 | -0.3685 | No |
| 20 | MAPT |  |  | 1627 | -0.270 | -0.4008 | No |
| 21 | NCS1 |  |  | 1647 | -0.273 | -0.4042 | No |
| 22 | PARP6 |  |  | 1649 | -0.274 | -0.4023 | No |
| 23 | LRRTM2 |  |  | 1691 | -0.278 | -0.4124 | No |
| 24 | ENO2 |  |  | 1707 | -0.281 | -0.4146 | No |
| 25 | NELFCD |  |  | 1724 | -0.283 | -0.4171 | No |
| 26 | GABRB3 |  |  | 1732 | -0.284 | -0.4169 | No |
| 27 | PEG3 |  |  | 1784 | -0.292 | -0.4298 | No |
| 28 | INO80C |  |  | 1868 | -0.300 | -0.4522 | No |
| 29 | DTD1 |  |  | 1925 | -0.308 | -0.4665 | No |
| 30 | ZNF22 |  |  | 1967 | -0.314 | -0.4763 | No |
| 31 | CLASP2 |  |  | 2005 | -0.318 | -0.4848 | No |
| 32 | TMEM108 |  |  | 2006 | -0.318 | -0.4822 | No |
| 33 | SCG3 |  |  | 2025 | -0.322 | -0.4850 | No |
| 34 | DDX39A |  |  | 2043 | -0.324 | -0.4875 | No |
| 35 | TRAP1 |  |  | 2055 | -0.326 | -0.4881 | No |
| 36 | ZNF711 |  |  | 2101 | -0.334 | -0.4989 | No |
| 37 | FSD1 |  |  | 2118 | -0.337 | -0.5010 | No |
| 38 | ADAM22 |  |  | 2159 | -0.344 | -0.5102 | No |
| 39 | RNF157 |  |  | 2160 | -0.345 | -0.5074 | No |
| 40 | ZNF195 |  |  | 2182 | -0.348 | -0.5109 | No |
| 41 | GLCCI1 |  |  | 2212 | -0.356 | -0.5167 | No |
| 42 | KIF1A |  |  | 2251 | -0.363 | -0.5251 | No |
| 43 | BEX2 |  |  | 2283 | -0.369 | -0.5314 | No |
| 44 | NET1 |  |  | 2290 | -0.370 | -0.5303 | No |
| 45 | TUBB4B |  |  | 2291 | -0.370 | -0.5273 | No |
| 46 | MSI2 |  |  | 2314 | -0.375 | -0.5308 | No |
| 47 | CRMP1 |  |  | 2324 | -0.377 | -0.5305 | No |
| 48 | QDPR |  |  | 2338 | -0.382 | -0.5313 | No |
| 49 | ST3GAL6 |  |  | 2346 | -0.383 | -0.5303 | No |
| 50 | MCM2 |  |  | 2349 | -0.383 | -0.5278 | No |
| 51 | ZWILCH |  |  | 2358 | -0.385 | -0.5271 | No |
| 52 | MSH6 |  |  | 2362 | -0.386 | -0.5248 | No |
| 53 | RALGDS |  |  | 2380 | -0.389 | -0.5268 | No |
| 54 | LEPROTL1 |  |  | 2437 | -0.399 | -0.5403 | No |
| 55 | ZNF738 |  |  | 2441 | -0.400 | -0.5380 | No |
| 56 | UCP2 |  |  | 2498 | -0.413 | -0.5514 | No |
| 57 | EVL |  |  | 2511 | -0.416 | -0.5517 | No |
| 58 | CSE1L |  |  | 2512 | -0.417 | -0.5483 | No |
| 59 | PLPPR5 |  |  | 2521 | -0.419 | -0.5473 | No |
| 60 | ATP6V1B2 |  |  | 2525 | -0.421 | -0.5448 | No |
| 61 | C14orf132 |  |  | 2577 | -0.434 | -0.5566 | No |
| 62 | ATCAY |  |  | 2583 | -0.435 | -0.5546 | No |
| 63 | TMOD2 |  |  | 2591 | -0.437 | -0.5531 | No |
| 64 | PHYHIPL |  |  | 2595 | -0.438 | -0.5505 | No |
| 65 | ZNF91 |  |  | 2608 | -0.441 | -0.5505 | No |
| 66 | GGCT |  |  | 2610 | -0.442 | -0.5472 | No |
| 67 | RNF165 |  |  | 2642 | -0.450 | -0.5529 | No |
| 68 | GDI1 |  |  | 2671 | -0.464 | -0.5575 | No |
| 69 | GDAP1 |  |  | 2675 | -0.465 | -0.5547 | No |
| 70 | FIGNL1 |  |  | 2679 | -0.465 | -0.5518 | No |
| 71 | FOXO3 |  |  | 2696 | -0.471 | -0.5528 | No |
| 72 | MCM6 |  |  | 2699 | -0.471 | -0.5496 | No |
| 73 | PRC1 |  |  | 2705 | -0.472 | -0.5472 | No |
| 74 | KNSTRN |  |  | 2722 | -0.478 | -0.5482 | No |
| 75 | EIF1B |  |  | 2747 | -0.485 | -0.5514 | No |
| 76 | RTN1 |  |  | 2775 | -0.496 | -0.5555 | No |
| 77 | NUF2 |  |  | 2781 | -0.497 | -0.5530 | No |
| 78 | RAB6B |  |  | 2814 | -0.507 | -0.5585 | Yes |
| 79 | IRS2 |  |  | 2821 | -0.509 | -0.5562 | Yes |
| 80 | ARL6IP1 |  |  | 2823 | -0.510 | -0.5523 | Yes |
| 81 | TMEM97 |  |  | 2834 | -0.514 | -0.5512 | Yes |
| 82 | SATB1 |  |  | 2870 | -0.527 | -0.5574 | Yes |
| 83 | FKBP4 |  |  | 2871 | -0.528 | -0.5531 | Yes |
| 84 | NMNAT2 |  |  | 2893 | -0.536 | -0.5551 | Yes |
| 85 | CDKN2C |  |  | 2903 | -0.544 | -0.5534 | Yes |
| 86 | ELAVL3 |  |  | 2924 | -0.559 | -0.5549 | Yes |
| 87 | OLA1 |  |  | 2946 | -0.569 | -0.5566 | Yes |
| 88 | ACOT7 |  |  | 2957 | -0.576 | -0.5549 | Yes |
| 89 | PBX3 |  |  | 2959 | -0.578 | -0.5505 | Yes |
| 90 | C3orf14 |  |  | 2962 | -0.580 | -0.5464 | Yes |
| 91 | KLHL13 |  |  | 2965 | -0.580 | -0.5424 | Yes |
| 92 | H1FX |  |  | 2968 | -0.581 | -0.5383 | Yes |
| 93 | SBK1 |  |  | 2970 | -0.582 | -0.5338 | Yes |
| 94 | GGH |  |  | 2975 | -0.585 | -0.5303 | Yes |
| 95 | OLFM1 |  |  | 2981 | -0.589 | -0.5270 | Yes |
| 96 | VRK1 |  |  | 2983 | -0.592 | -0.5226 | Yes |
| 97 | DKK1 |  |  | 2992 | -0.596 | -0.5201 | Yes |
| 98 | PPP1R9A |  |  | 2994 | -0.599 | -0.5156 | Yes |
| 99 | ALK |  |  | 3014 | -0.610 | -0.5164 | Yes |
| 100 | PNMA2 |  |  | 3022 | -0.615 | -0.5135 | Yes |
| 101 | LSM3 |  |  | 3037 | -0.625 | -0.5126 | Yes |
| 102 | EEF1A2 |  |  | 3044 | -0.628 | -0.5094 | Yes |
| 103 | GAL |  |  | 3046 | -0.631 | -0.5046 | Yes |
| 104 | RBBP8 |  |  | 3053 | -0.637 | -0.5012 | Yes |
| 105 | CYFIP2 |  |  | 3063 | -0.645 | -0.4987 | Yes |
| 106 | CCND1 |  |  | 3072 | -0.655 | -0.4958 | Yes |
| 107 | ZNF704 |  |  | 3086 | -0.665 | -0.4943 | Yes |
| 108 | ANP32A |  |  | 3090 | -0.666 | -0.4898 | Yes |
| 109 | RBP1 |  |  | 3091 | -0.667 | -0.4844 | Yes |
| 110 | DPYSL2 |  |  | 3104 | -0.680 | -0.4825 | Yes |
| 111 | DACH1 |  |  | 3112 | -0.686 | -0.4791 | Yes |
| 112 | LSM4 |  |  | 3115 | -0.688 | -0.4741 | Yes |
| 113 | SHC3 |  |  | 3118 | -0.690 | -0.4691 | Yes |
| 114 | RFC4 |  |  | 3129 | -0.701 | -0.4665 | Yes |
| 115 | SCG2 |  |  | 3130 | -0.703 | -0.4608 | Yes |
| 116 | GMNN |  |  | 3140 | -0.719 | -0.4577 | Yes |
| 117 | KIF15 |  |  | 3142 | -0.723 | -0.4521 | Yes |
| 118 | FZD3 |  |  | 3147 | -0.727 | -0.4474 | Yes |
| 119 | TUBB3 |  |  | 3152 | -0.731 | -0.4427 | Yes |
| 120 | CCSAP |  |  | 3156 | -0.737 | -0.4377 | Yes |
| 121 | PRIM1 |  |  | 3160 | -0.739 | -0.4326 | Yes |
| 122 | DPYSL5 |  |  | 3164 | -0.746 | -0.4275 | Yes |
| 123 | DNAJC9 |  |  | 3167 | -0.747 | -0.4220 | Yes |
| 124 | AP1S2 |  |  | 3206 | -0.789 | -0.4270 | Yes |
| 125 | BIRC5 |  |  | 3208 | -0.793 | -0.4209 | Yes |
| 126 | CXCR4 |  |  | 3217 | -0.803 | -0.4168 | Yes |
| 127 | FHOD3 |  |  | 3218 | -0.806 | -0.4103 | Yes |
| 128 | UBE2C |  |  | 3225 | -0.812 | -0.4055 | Yes |
| 129 | NCAN |  |  | 3228 | -0.816 | -0.3995 | Yes |
| 130 | KIF5C |  |  | 3232 | -0.823 | -0.3938 | Yes |
| 131 | NUSAP1 |  |  | 3237 | -0.825 | -0.3883 | Yes |
| 132 | FOXM1 |  |  | 3238 | -0.826 | -0.3816 | Yes |
| 133 | GRIA2 |  |  | 3258 | -0.862 | -0.3804 | Yes |
| 134 | CCP110 |  |  | 3262 | -0.870 | -0.3742 | Yes |
| 135 | CDCA5 |  |  | 3273 | -0.885 | -0.3701 | Yes |
| 136 | TFAP2B |  |  | 3274 | -0.886 | -0.3629 | Yes |
| 137 | KLHL23 |  |  | 3287 | -0.907 | -0.3592 | Yes |
| 138 | RANBP1 |  |  | 3290 | -0.911 | -0.3524 | Yes |
| 139 | CDKN3 |  |  | 3293 | -0.916 | -0.3456 | Yes |
| 140 | HMGA1 |  |  | 3299 | -0.931 | -0.3396 | Yes |
| 141 | TMTC4 |  |  | 3303 | -0.936 | -0.3329 | Yes |
| 142 | TCEAL7 |  |  | 3306 | -0.939 | -0.3259 | Yes |
| 143 | GNG4 |  |  | 3323 | -0.971 | -0.3229 | Yes |
| 144 | UBE2T |  |  | 3330 | -0.979 | -0.3167 | Yes |
| 145 | IGSF3 |  |  | 3331 | -0.981 | -0.3088 | Yes |
| 146 | ASRGL1 |  |  | 3333 | -0.984 | -0.3012 | Yes |
| 147 | KIF21A |  |  | 3334 | -0.986 | -0.2932 | Yes |
| 148 | MYBL2 |  |  | 3340 | -1.006 | -0.2865 | Yes |
| 149 | PBK |  |  | 3354 | -1.046 | -0.2820 | Yes |
| 150 | DPYSL3 |  |  | 3377 | -1.119 | -0.2795 | Yes |
| 151 | TAGLN3 |  |  | 3378 | -1.121 | -0.2705 | Yes |
| 152 | ATP6V0E2 |  |  | 3380 | -1.133 | -0.2616 | Yes |
| 153 | MCM7 |  |  | 3384 | -1.147 | -0.2532 | Yes |
| 154 | EML6 |  |  | 3393 | -1.197 | -0.2460 | Yes |
| 155 | CENPU |  |  | 3398 | -1.230 | -0.2372 | Yes |
| 156 | NCAM1 |  |  | 3416 | -1.284 | -0.2319 | Yes |
| 157 | PKIA |  |  | 3417 | -1.286 | -0.2215 | Yes |
| 158 | MYEF2 |  |  | 3419 | -1.290 | -0.2114 | Yes |
| 159 | RRM2 |  |  | 3420 | -1.297 | -0.2009 | Yes |
| 160 | MAP2 |  |  | 3422 | -1.305 | -0.1907 | Yes |
| 161 | HES6 |  |  | 3431 | -1.366 | -0.1820 | Yes |
| 162 | CXADR |  |  | 3435 | -1.390 | -0.1717 | Yes |
| 163 | AKAP12 |  |  | 3444 | -1.501 | -0.1619 | Yes |
| 164 | GAP43 |  |  | 3445 | -1.504 | -0.1498 | Yes |
| 165 | CELF2 |  |  | 3464 | -1.718 | -0.1413 | Yes |
| 166 | TUBB2B |  |  | 3465 | -1.731 | -0.1273 | Yes |
| 167 | SOX11 |  |  | 3469 | -1.784 | -0.1138 | Yes |
| 168 | INSM1 |  |  | 3483 | -1.929 | -0.1021 | Yes |
| 169 | ASCL1 |  |  | 3484 | -1.937 | -0.0864 | Yes |
| 170 | CADM1 |  |  | 3485 | -1.943 | -0.0707 | Yes |
| 171 | BEX1 |  |  | 3490 | -2.151 | -0.0545 | Yes |
| 172 | NRCAM |  |  | 3495 | -2.259 | -0.0374 | Yes |
| 173 | CKB |  |  | 3497 | -2.376 | -0.0185 | Yes |
| 174 | DCX |  |  | 3505 | -2.772 | 0.0018 | Yes |
Table: GSEA details [plain text format]

  

Fig 2: VANGRONINGEN\_ADRN: Random ES distribution      
 Gene set null distribution of ES for **VANGRONINGEN\_ADRN**
